# Fitting a vaccine into the HIV prevention landscape

**DOI:** 10.1002/jia2.25792

**Published:** 2021-11-21

**Authors:** Anthony S. Fauci, Carl W. Dieffenbach, François Dabis

**Affiliations:** ^1^ Office of the Director National Institute of Allergy and Infectious Diseases National Institutes of Health Bethesda Maryland USA; ^2^ Division of AIDS National Institute of Allergy and Infectious Diseases National Institutes of Health Bethesda Maryland USA; ^3^ Centre de Recherche INSERM U.1219 Bordeaux Population Health Institut de SantePublique Epidemiologie et Developement (ISPED) Universite de Bordeaux Bordeaux France

**Keywords:** antibody‐mediated prevention, broad neutralizing antibodies, HIV treatment and prevention, HIV vaccine efficacy, long‐acting, pre‐exposure prophylaxis

The development of highly effective treatment and prevention strategies for HIV has been a significant scientific achievement over the past 25 years. Although progress has been made in the global access of antiretroviral treatment (ART), the UNAIDS 2020 global targets were not achieved because of structural and individual barriers. Therefore, the global targets are being revised for the 2025–2030 timeframe [1]. In this article, we will discuss the current state of HIV treatment and prevention and describe the research agenda that will help advance the next generation of strategies. In the context of this rapidly evolving prevention and treatment landscape, the levels of efficacy expected from an HIV vaccine increase. While using some of the lessons learned from SARS‐CoV‐2 vaccines, we will also point out that a safe, efficacious and durable HIV vaccine is achievable and remains a worthy goal.

As of June 2020, global ART coverage was estimated at 26 million people living with HIV (PLWH), two‐thirds of the universal treatment target. Current estimates of adherence suggest that 59% of PLWH are virus‐free worldwide and thus can expect relatively normal life spans [2]. Furthermore, global endorsement of “undetectable equals untransmissible”, also known as U = U, makes ART the most potent and widely used preventive approach today [3]. The first long‐acting injectable ART regimen delivered via monthly injection has been approved by regulatory authorities. Additional long‐acting formulations are in development, including several containing broadly neutralizing antibodies (bNAbs) that could potentially extend the injection time from once every 6 months to once a year. Simplified delivery of long‐acting combinations will result in both greater population coverage and durable virologic suppression. Furthermore, greater population coverage with these potent, durable regimens will boost the role of treatment as prevention in helping to control the global HIV pandemic. With these significant periods of time between doses, long‐acting ART may well soon become a preferred option for treatment of PLWH.

Except for barrier methods and adult medical male circumcision, all successful HIV prevention methods rely on antiretroviral drugs [4, 5]. The hypothesis has been that if there is a sufficient concentration of drug blocking HIV replication in the exposed tissues, then virus cannot establish infection, allowing the individual to remain HIV‐free. Studies of different drug regimens of oral pre‐exposure prophylaxis (PrEP) have shown that with adherence, protection from acquisition of HIV is remarkably high, greater than 95% and consistent across populations [6]. However, with only approximately a million PrEP users in 70 countries, the full impact of this powerful preventive tool is far from being fully achieved [6]. A major challenge facing PrEP‐based HIV prevention has been the lack of sustained use by individuals who would gain the most benefit from the intervention. In studies comparing methods to improve PrEP rollout, high PrEP uptake and initiation was achieved at onset, but was then followed by significant levels of PrEP discontinuation [7]. One strategy to address adherence challenges has been the development of long‐acting PrEP. In 2020, PrEP trials of an injectable drug, administered every 8 weeks, demonstrated superior efficacy compared to the standard daily oral PrEP among at‐risk women and men. This significant achievement will soon lead to drug approval and implementation [8, 9]. As with therapy, new agents for PrEP are moving forward in the evaluation process that could increase the amount of time between doses to once or twice a year. This could lead to increased adherence, with significant improvements in effectiveness and less burden on the health system, compared to daily pills.

In addition to antivirals, bNAbs are being explored as prevention modalities. The recently completed Antibody Mediated Prevention (AMP) trials demonstrated that the protection from infection is governed by the sensitivity of the virus to the bNAbs [10]. In addition to advancing important options for treatment and prevention of HIV infection, bNAbs also serve as a window into the levels of activity a future HIV vaccine will need to be able to trigger [11].

New medications for HIV treatment are also being evaluated for HIV prevention. Based upon the properties of each new drug, a variety of dosing options are under consideration – from weekly to once or twice a year, delivered by pill or injection. Currently, biomedical HIV prevention modalities include daily pills, monthly vaginal rings and injections every 8 weeks. The next generation of bNAbs have also been engineered to have optimized neutralization breadth and longer half lives *in vivo* [12]. Optimized cocktails of two and three antibodies are in development and, based upon the AMP results, have a high probability of success.

Using bNAbs as a guide, it is reasonable to set an aspirational goal for vaccine efficacy that is significantly higher than previously proposed. Progress is being made in defining the requirements for induction of bNAbs via immunization and using new tools and technologies to deliver specifically engineered vaccines it should be possible to reproducibly and durably trigger production of antibodies from at least three bNAb families. Based upon the activity of bNAbs, it may be possible to achieve a level of efficacy in the 75–85% range.

Importantly, there are two HIV vaccine efficacy trials ongoing, HVTN 705, Imbokodo, and HVTN 706, Mosaico [13, 14]. As an approach to facilitate and improve HIV vaccine discovery, there are lessons we can learn from SARS‐CoV‐2 vaccines [15]. As research focused on HIV vaccine discovery moves forward, there are many new tools and platforms we can adapt from the SARS‐CoV‐2 vaccines, including the utilization of various platform technologies and the development of optimally conformed immunogens. Acknowledging that the natural immunity that is induced by current HIV vaccines or HIV infection does not provide protection, the new tools that SARS CoV‐2 can provide help to iterate vaccine strategies faster [15, 16]. Addressing the fundamental challenges of HIV vaccine development, combined with operationalizing new concepts, and fostering significant industry engagement will be the research focus for the next decade. The research described throughout this Journal Supplement will play a critical role to achieve our 2030 targets of durable control of the HIV pandemic.

## COMPETING INTERESTS

The authors have no competing interests or holdings.

## AUTHORS' CONTRIBUTIONS

First draft was assembled by CD. AF and FD edited the drafts and CD assembled the final submission.

## FUNDING

None.
